# A120 A HUMAN-CENTERED DESIGN APPROACH TO THE DEVELOPMENT OF A VACCINE IMPLEMENTATION STRATEGY IN PATIENTS WITH INFLAMMATORY BOWEL DISEASE

**DOI:** 10.1093/jcag/gwad061.120

**Published:** 2024-02-14

**Authors:** F Zhou, J Robar, M Stewart, J Jones

**Affiliations:** Dalhousie University, Halifax, NS, Canada; Dalhousie University, Halifax, NS, Canada; Dalhousie University, Halifax, NS, Canada; Dalhousie University, Halifax, NS, Canada

## Abstract

**Background:**

Vaccination uptake amongst patients with inflammatory bowel disease (IBD) remains suboptimal. Our previous study assessed perceived barriers and solutions related to implementation of evidence-based guidelines for vaccine preventable illness (VPI). Barriers included limited time, lack of access to a family physician, and incomplete understandings of coverage/access to vaccines.

**Aims:**

The aim of this study was to use human-centered design (HCD) to design a vaccine implementation program in patients with IBD.

**Methods:**

The HCD approach consisted of multiple phases. Phase 1 (discovery and define) consisted of semi-structured interviews of healthcare providers. Phase 1 also included the development of a context-specific patient journey map of the processes involved in accessing vaccines. This map was developed in collaboration with a multidisciplinary IBD team. Phase 2 involved identifying common barriers and facilitators through thematic analysis and journey mapping. In Phase 3, these analyses were used to design an implementation strategy for evidence-based management of VPI in the local health system context.

**Results:**

Twelve interviews (including 11 gastroenterologists and one IBD nurse practicing in Nova Scotia and New Brunswick) were conducted. Mean participant age was 45.1 years, with 63.6% identifying practice in an urban/academic setting compared to a rural/community setting (36.4%). Lack of access to a family physician, limited time, vaccine hesitancy, and incomplete understanding of coverage/access to vaccines were among the barriers identified. Using facilitator themes as well as the journey mapping process, a proposal for an early implementation strategy prototype was designed. Prototype development involved collaborative partnership with a third-party patient support program to overcome various barriers identified in our surveys.

**Conclusions:**

Barriers to implementation of evidence-based guidelines for management of VPI are well documented. The use of HCD to design an effective implementation strategy that is sensitive to the local healthcare context holds potential to increase access to and uptake of high quality, evidence-based VPI management. In future studies, the implementation-effectiveness of the implementation strategy (model prototype) will be evaluated and compared to standard of care.

**Funding Agencies:**

None

